# Resveratrol Ameliorates High Altitude Hypoxia-Induced Osteoporosis by Suppressing the ROS/HIF Signaling Pathway

**DOI:** 10.3390/molecules27175538

**Published:** 2022-08-28

**Authors:** Changqing Yan, Zirou Wang, Weili Liu, Lingling Pu, Ran Li, Chongyi Ai, Hongbao Xu, Baoyi Zhang, Tianhui Wang, Xiangyu Zhang, Zhaoli Chen, Xinxing Wang

**Affiliations:** 1 Tianjin Institute of Environmental and Operational Medicine, Tianjin 300050, China; 2 School and Hospital of Stomatology, Tianjin Medical University, Tianjin 300070, China

**Keywords:** Resveratrol, osteoblast, osteoclast, osteoporosis, HIF-1α

## Abstract

Hypoxia at high-altitude leads to osteoporosis. Resveratrol (RES), as an antioxidant, has been reported to promote osteoblastogenesis and suppress osteoclastogenesis. However, the therapeutic effect of RES against osteoporosis induced by high-altitude hypoxia remains unclear. Thus, this study was intended to investigate the potential effects of RES on high-altitude hypoxia-induced osteoporosis both in vivo and in vitro. Male Wistar rats were given RES (400 mg/kg) once daily for nine weeks under hypoxia, while the control was allowed to grow under normoxia. Bone mineral density (BMD), the levels of bone metabolism-related markers, and the changes on a histological level were measured. Bone marrow-derived mesenchymal stem cells (BMSCs) and RAW264.7 were incubated with RES under hypoxia, with a control growing under normoxia, followed by the evaluation of proliferation and differentiation. The results showed that RES inhibited high-altitude hypoxia-induced reduction in BMD, enhanced alkaline phosphatase (ALP), osteocalcin (OCN), calcitonin (CT) and runt-related transcription factor 2 (RUNX2) levels, whereas it reduced cross-linked carboxy-terminal telopeptide of type I collagen (CTX-I) levels and tartrate-resistant acid phosphatase (TRAP) activity in vivo. In addition, RES attenuated histological deteriorations in the femurs. In vitro, RES promoted osteoblastogenesis and mineralization in hypoxia-exposed BMSCs, along with promotion in RUNX2, ALP, OCN and osteopontin (OPN) levels, and inhibited the proliferation and osteoclastogenesis of RAW264.7. The promotion effects of RES on osteoblastogenesis were accompanied by the down-regulation of reactive oxygen species (ROS) and hypoxia inducible factor-1α (HIF-1α) induced by hypoxia. These results demonstrate that RES can alleviate high-altitude hypoxia-induced osteoporosis via promoting osteoblastogenesis by suppressing the ROS/HIF-1α signaling pathway. Thus, we suggest that RES might be a potential treatment with minimal side effects to protect against high-altitude hypoxia-induced osteoporosis.

## 1. Introduction

As a chronic mountain sickness, high-altitude osteoporosis continuously puts people at high-altitudes into trouble [1,2]. The acute and life-threatening clinical manifestations of high-altitude cerebral edema and pulmonary edema have attracted the attention of many scholars, but the research on chronic diseases induced by high-altitude such as osteoporosis has stagnated [3]. The adverse impacts of high-altitude hypoxia on bone metabolism have been recognized in the past few decades, while the pathology of high-altitude osteoporosis remains unclear and controversial [4,5,6]. Hypobaric hypoxia is the most important environmental factor affecting health [7]. With an increase in altitude and decrease in oxygen, bone health continues to deteriorate [8]. Changes occur not only in the early markers of osteoblast and osteoclast in the blood, but also in the morphology and structure of bone tissue [9].

Bone remodeling is regulated by a dynamic balance between bone anabolism and bone catabolism to ensure bone health, in which osteoblasts, osteoclasts and bone marrow mesenchymal stem cells take part [10]. Under hypoxia conditions, oxidative stress results in the upward release of reactive oxygen species (ROS), exerting effect on bone remodeling [11,12,13,14]. As a downstream factor and inducer of ROS, hypoxia inducible factor-1 (HIF-1α) plays a pivotal role in bone remodeling and effectively regulates the physiological activities of various bone-related cells [15,16]. Many scholars have paid attention to the relationship between the changes of bone metabolism induced by the pathological hypoxia and HIF-1α, but the relationship between the changes of bone metabolism induced by the chronic hypoxia environment and HIF-1α has not been explored. Previous studies have shown that HIF-1α overexpression can increase the formation of H-type blood vessels, and the coupling of osteogenesis and angiogenesis can ameliorate postmenopausal osteoporosis [17]. On the other hand, there is the finding that the OVX rats, which have an osteoporosis-like performance, have a higher level of HIF-1α. After treatment with drugs, the osteoporosis was alleviated and the level of HIF-1α decreased [18]. Therefore, the role of HIF-1α in osteoporosis, especially in high-altitude osteoporosis, remains controversial and unclear.

Considering hypoxia as a cause of high-altitude-induced osteoporosis, drugs against oxidative stress have become a treatment strategy for high-altitude osteoporosis [19,20]. Resveratrol (RES) has long been in clinical use in European and American countries. Extracted from Polygonum cuspidatum, Cassia cuspidatum, grapes and other plants, this substance has many functions, such as anti-oxidation, anti-tumorand anti-cardiovascular diseases [21,22]. According to reports, RES has a phytoestrogen-like effect, which can regulate bone cell metabolism and bone turnover [23,24]. At the same time, it can effectively reduce the oxidative stress caused by body aging and loss of mass due to the lack of estrogen after menopause [25,26]. However, despite accumulating data on the effect of RES on osteoporosis, the effect of RES on high-altitude osteoporosis is not clearly defined; thus, we investigated the effect of RES on this disorder.

## 2. Materials and Methods

### 2.1. Reagents

Resveratrol (RES, C_14_H_12_O_3_, CAS NO.: 501-36-0) was purchased from Beyotime (Shanghai, China). Sodium carboxymethyl cellulose (Sigma-Aldrich, St. Louis, MO, USA) was used to suspend resveratrol for animal experiments and dimethylsulfoxide (DMSO, Solarbio, Beijing, China) to dissolve resveratrol for cell experiments.

### 2.2. BMSCs Extraction, Culture, and Identification

Bone marrow-derived mesenchymal stem cells (BMSCs) were obtained from the bone marrow of 2-week-old male Wistar rats. Briefly, Wistar rats were sacrificed and immersed in 75% ethanol before femur was isolated. The marrow cavity was repeatedly washed with Minimum Essential Medium-α (α-MEM, Procell, Wuhan, China) after the rat bones had been washed and the epiphysis of long bones had been removed. The fluids were collected and inoculated in a culture dish. For identification, BMSCs were trypsinized and washed by using phosphate-buffered saline (PBS). The cells were subsequently resuspended to a density of 1 × 10^6^ cells/mL. Suspensions were distributed to Eppendorf (EP) tubes, which was 200 µL per tube. Monoclonal antibodies against CD29 (FITC-conjugated, Gibco, New York, NY, USA), CD45 (FITC-conjugated, Elabscience, Wuhan, China), CD90 (FITC-conjugated, Elabscience, Wuhan, China) and CD11 (FITC-conjugated, Biolegend, San Diego, CA, USA) were then added to the EP tubes. After incubation, washing and resuspending, the cell phenotype was detected by a flow cytometer (BD Biosciences, Franklin Lakes, NJ, USA).

### 2.3. Assessment of Cell Viability

BMSCs were cultured in a hypoxic workstation for various durations (0 h, 12 h, 24 h and 48 h). After culture, CCK8 solution (Invigentech, San Diego, CA, USA) was added into the medium. The OD was read by a microplate reader (Molecular Devices, Sunnyvale, CA, USA) after incubation. Next, BMSCs were grouped into four experimental groups: control group, hypoxia group, low-dose RES group (L-RES, 0.1 μM) and high-dose RES group (H-RES, 1 μM). The control group was cultured under normoxia (21% O_2_) all the time, while the other three groups were cultured under hypoxia (1% O_2_). After treatment, CCK8 assays were carried out as above.

Proliferation of RAW264.7 under hypoxia and the effect of RES were measured in the same way as for BMSCs.

### 2.4. Osteoblast Differentiation

BMSCs were plated on 24 well plates or 6 well plates. Osteogenic induction medium (α-MEM containing 10% FBS, 1 μM dexamethasone, 10 mM β-glycerophosphate, 50 mg/L ascorbic acid and 1% penicillin-streptomycin) was applied to induce in vitro osteoblastogenesis of BMSCs. Cells were grouped into four experimental groups: control group, hypoxia group, low-dose RES group (L-RES group, 0.1 μM) and high-dose RES group (H-RES group, 1 μM). During osteogenic differentiation, the control group was cultured under normoxia (21% O_2_), while the other three groups were cultured under hypoxia (1% O_2_). Cell culture under hypoxia was carried out in anaerobic workstations (in vivo 2400, Ruskinn Technologies, Bridgend South Wales, UK) with oxygen-deprived atmosphere and temperature controlled at 37 °C. The dosage of RES and the concentration of O_2_ used to mimic hypoxia was determined according to the literature [27,28].

### 2.5. Alizarin Red S (ARS) Staining

BMSCs were subjected to induction for osteoblastogenesis for 20 days. Before staining, cells were rinsed and fixed, and 1% alizarin red S staining solution (pH 4.2, Solarbio, Beijing, China) was used for staining. An inverted microscope was used to observe calcification nodules and capture images (Leica, Weztlar, Germany, magnification 100×).

### 2.6. ALP Staining

BMSCs were subjected to induction for osteoblastogenesis for 7 days. Before staining, cells were rinsed and fixed. After being washed three times, a BCIP/NBT ALP Color Development Kit (Beyotime, Shanghai, China) was used to stain the cells. A camera was used to capture images. Image J software was used to quantify the expression and activity of ALP.

### 2.7. Real-Time Polymerase Chain Reaction (RT-PCR)

Total ribonucleic acid (RNA) in BMSCs was extracted by using a UNlQ-10 Column Trizol Total RNA Isolation Kit (Sangon, Shanghai, China) and it was subjected to reverse transcription. The extracted complementary deoxyribonucleic acid (cDNA) was applied for the polymerase chain reaction (PCR) by using the SYBR Green method (Biorad, Hercules, CA, USA). Primer sequences were as follows:

OCN forward, 5′-TTGAGCTCACACACCTCCCTGT-3′
OCN reverse, 5′-TGCAAAGCCCAGCGACTCT-3′
OPN forward, 5′-TTGATAGCCTCATCGGACTCCTG-3′
OPN reverse, 5′- GCCGAGGTGATAGCTTGGCTTA-3′
ALP forward, 5′-TGACCACCACTCGGGTGAA-3′
ALP reverse, 5′-GCATCTCATTGTCCGAGTACCA-3′
RUNX2 forward, 5′-CAAGTGGCCAGGTTCAACGA-3′
RUNX2 reverse, 5′-GGGACCTGCCACTGTCACTGTAATA-3′
β-actin forward, 5′-CCCCATTGAACACGGCATTG-3′
β-actin reverse, 5′-TCATAGAAGAGAGTCCTGGGTCA-3′.

### 2.8. Assessment of ROS

The ROS level in BMSCs was measured by the Reactive Oxygen Species Assay kit (Beyotime, Shanghai, China). Briefly, the cells were grouped and treated with RES of different concentrations as above for 48 h under hypoxia. After treatment, the cells were rinsed, followed by incubation with 1 mM DCHF-DA away from light. The cells were then trypsinized and washed again before being resuspended in PBS for the detection, which was conducted on a BD flow cytometer using Diva software.

### 2.9. Western Blotting

Radioimmunoprecipitation assay (RIPA) lysis buffer (Solarbio, Beijing, China) was used to extract total protein from BMSCs. Quantification of proteins samples was performed by bicinchoninic acid (BCA) method (Solarbio, Beijing, China). After denaturation, the protein samples were then subjected to electrophoresis and transferred on a polyvinylidene difluoride (PVDF) membranes (Millipore, USA). After electrotransfer, skim milk was used to block the membrane, followed by addition of primary antibodies (anti-HIF-1α and anti-PHD2, Abcam) for overnight incubation. Then, the secondary antibodies were used to probe the proteins. Bands were exposed by the enhanced chemiluminescence (ECL) method (General Electric, Boston, MA, USA) and analysis of bands’ gray value was achieved by Image J Software.

### 2.10. Osteoclast Differentiation

RAW264.7 was grouped as above: control group, hypoxia group, L-RES group (0.1 μM) and H-RES group (1 μM). The control group was cultured under normoxia (21% O_2_), while other three groups were cultured under hypoxia (1% O_2_). Induction was performed by adding the receptor activator for nuclear factor-κB ligand (RANKL, 100 ng/mL) into the medium.

### 2.11. TRAP Staining

To measure the osteoclastogenesis of RAW264.7 under hypoxia and the effect of RES on it, the cells were plated and grouped as above. After 7 days’ induction by adding RANKL (100 ng/mL) into the medium, the level of osteoclast was determined by TRAP staining. The staining was conducted according to the kits (Sigma-Aldrich, St. Louis, MO, USA).

### 2.12. Experimental Animals

Male Wistar rats (3 months old, weighing ~330 g) were bred under normoxia unless otherwise stated. All the animals were given water and a pelleted diet ad libitum. Rats were divided into three groups at random (ten rats per group): the control group, hypoxia exposure group, hypoxia exposure with RES group. The rats in the hypoxia exposure with RES group were administrated with RES of 400 mg/kg for 9 weeks according to the previous research [29]. All the groups except the control group were placed in a hypobaric chamber (Hongyuan Oxygen Industry, Yantai, China) to imitate hypoxia at an altitude of 6000 m for 20 h (12:30 p.m.–8:30 a.m.) per day over 9 weeks. All rats were administered 0.5% sodium carboxymethyl cellulose and drugs suspended in equal volumes of saline orally through gavage. After 9 weeks, the rats were killed through euthanasia by phlebotomy after injection of anesthesia. Serum was obtained and stored at −80 °C until measurement. The femurs were taken out and dissected until soft tissue was moved away, followed by fixation in 4% paraformaldehyde solution before micro-computed tomography (Micro-CT) analysis, histomorphometry observation and immunohistochemical staining. All procedures relating to animal care and use were approved by the Ethics Review Committee of the Institute of Environmental and Operational Medicine (IACUC of AMMS-04-2020-040).

### 2.13. Dual Energy X-Ray Absorptiometry Analysis

At the ninth week, bone mineral density (BMD) analysis of rats was carried out under a small animal anesthesia machine (RWD Life Science, Shenzhen, China). A dual-energy X-ray imaging system (InAlyzer, Seoul, Korea; 55 KeV and 80 Kev) was used to measure the BMD. After the rat body became soft, it was transferred to a dual-energy X-ray absorptiometry in a state of continuous anesthesia. The ROI tool was used to delineate the shape of the right femur, and the bone density in the ROI region of the rat was analyzed using the bone densitometer software.

### 2.14. Micro-Computed Tomography (Micro-CT) Analysis

The structure of the femur was analyzed by using Micro-CT (Bruker, Karlsruhe, Germany). The femurs were scanned with a voltage of 85 kV and a current of 200 mA, at 10 mm under the growth plate. Three-dimensional analyses were carried out by using the following software packages: 3DVox to reconstruct image, CT Vol to visualize three-dimensional model, and CT An to analyze data. Bone parameters were indicated as the trabecular bone volume per tissue volume (BV/TV), trabecular number (Tb. N), and trabecular separation (Tb. Sp).

### 2.15. Hematoxylin-Eosin (HE) and Immunohistochemical Staining

After the micro-CT analysis, the fresh femurs were dissected and decalcified for 21 days. After decalcification, the femurs were embedded in paraffin and sagittally sectioned (5 μm). HE staining was conducted for histological evaluation. To determine the number and surface characteristics of osteoclasts, tartrate-resistant acid phosphatase (TRAP) staining was performed. The expression of runt-related transcription factor 2 (RUNX2), HIF-1α and prolyl hydroxylase (PHD2) were measured to evaluate HIF-1α’s effect on high-altitude hypoxia-induced osteoporosis. The sections were observed by using a microscope, and a digital camera was used to capture images. The number of TRAP + multinucleated cells (three or more nuclei) were counted as osteoclasts (magnification 100×). The expressions of RUNX2, HIF-1α and PHD2 were quantified by image J software (magnification 400×).

### 2.16. Serum Biochemical Analysis

Alkaline phosphatase (ALP), osteocalcin (OCN), calcitonin (CT) and cross-linked carboxy-terminal telopeptide of type I collagen (CTX-I) in serum were measured respectively by enzyme-linked immunosorbent assay (ELISA) kits according to their protocols (Jianglai biotech, Shanghai, China).

### 2.17. Assessment of Antioxidant Capacity

The rats’ serum was collected as mentioned above. Serum level of malondialdehyde (MDA), activity of superoxide dismutase (SOD) and catalase (CAT), and total antioxidation capacity (T-AOC) were measured respectively with kits according to the kits’ instructions (Nanjing Jiancheng, Nanjing, China). Oxidation levels were reflected by MDA levels, and the thiobarbituric acid (TBA) method was used to quantify that. After the preparation of the samples and standards, the OD value was measured at 532 nm. SOD, CAT activity and T-AOC were quantified to indicate the intracellular anti-oxidation conditions. The SOD activity was investigated by quantifying the levels of formazan dye, after conduction according to the kit, OD values were measured at 450 nm. The CAT activity was evaluated by using a CAT assay kit which determined the enzyme’s activity according to the remaining H_2_O_2_. T-AOC was determined by using the T-AOC assay kit, which evaluated the total antioxidant by detecting the generation of ABTS+.

The cells were divided into groups and treated as above. After treatment, the cells were harvested. Ultrasonication was used to crush cells, followed by centrifugation. Intracellular MDA, SOD, CAT and T-AOC were measured as above.

### 2.18. Statistical Analysis

One-way ANOVA was used for analyzing differences between the groups with PRISM, version 8.0 (GraphPad Software, San Diego, CA, USA). All data are shown as mean ± SD for each group. It was considered to be statistically different only when *p* < 0.05.

## 3. Results

### 3.1. RES Promoted Cell Viability and Osteoblastogenesis in BMSCs under Hypoxia

**Figure 1:**
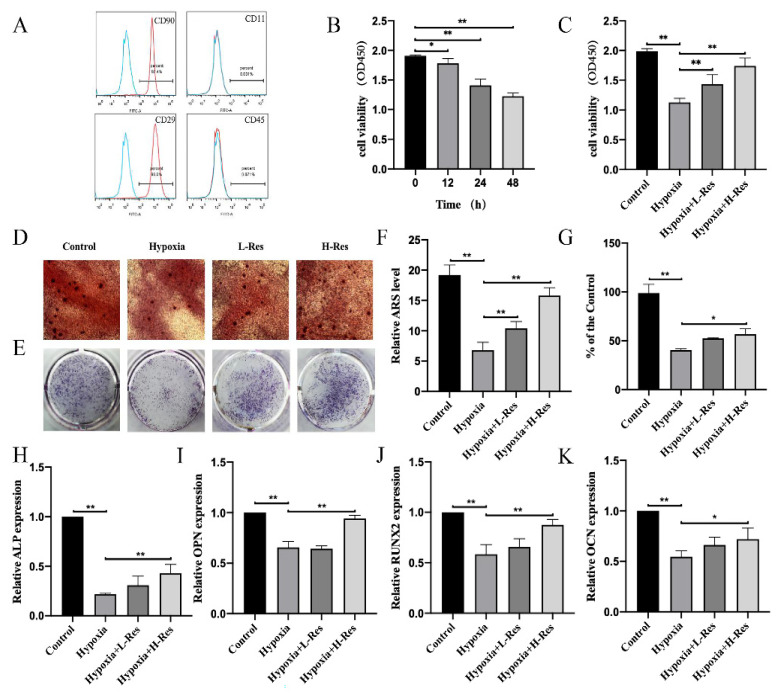
RES promoted the cell viability and osteoblastogenesis in BMSCs under hypoxia. (**A**) Flow cytometry demonstrated that BMSCs presented high expressions of CD29 and CD90, and low expressions of CD11 and CD45. (**B**) The viability of BMSCs under hypoxia was measured. (**C**) The viability of BMSCs from control group, hypoxia group, L-RES group and H-RES group was detected. (**D**) ARS staining results of BMSCs from control group, hypoxia group, L-RES group and H-RES group. (**E**) ALP staining results of BMSCs from control group, hypoxia group, L-RES group and H-RES group.(**F**,**G**) quantification for %area. The mRNA expression level of (**H**) ALP, (**I**) OPN, (**J**) RUNX2, (**K**) OCN were measured by RT-PCR. Values were expressed as mean ± SD. * *p* < 0.05, ** *p* < 0.01; all the assays were repeated more than three times.

The isolated BMSCs showed high expressions of CD29 (98.0%) and CD90 (97.4%) but low expressions of CD11 (0.031%) and CD45 (0.071%) (Figure 1A).

To study hypoxia’s effect on the proliferation of BMSCs, a CCK8 assay was used. As the results showed, the cell viability decreased under hypoxia in a time-dependent manner (Figure 1B; *p* < 0.05). In order to investigate whether RES could rescue the low proliferation caused by hypoxia, the cells were divided into four groups: control group (21% O_2_), hypoxia group (1% O_2_), L-RES group (1% O_2_ with 0.1 μM RES) and H-RES group (1% O_2_ with 1 μM RES). The results showed that RES could improve cell viability under hypoxia in a dose-dependent manner (Figure 1C; *p* < 0.01).

Next, the effect of hypoxia on osteoblastogenesis and the regulation of RES was investigated. ARS and ALP staining were used to evaluate the osteoblast activity of BMSCs. ARS staining results showed that the mineralized bone nodules formed less under hypoxia compared to the control group, while RES promoted that formation in a dose-dependent manner (Figure 1D). The level of mineralized bone nodules indicated by ARS was shown in Figure 1F (*p* < 0.01). Consistently, ALP staining demonstrated that hypoxia decreased the ALP activity under hypoxia compared to the control group, while RES worked in a dose-dependent manner (Figure 1E) and their relative level was shown in Figure 1G (*p* < 0.05).

RT-PCR was also used to evaluate osteoblastogenesis at the gene expression level. Results showed the downregulation of four bone-formation related genes, ALP, RUNX2, OPN and OCN, under the hypoxia relative to control group, while RES improved the expression of these genes in a dose-dependent manner (Figure 1H–K; *p* < 0.05).

### 3.2. RES Inhibited Cell Viability and Osteoclastogenesis in RAW 264.7 under Hypoxia

**Figure 2:**
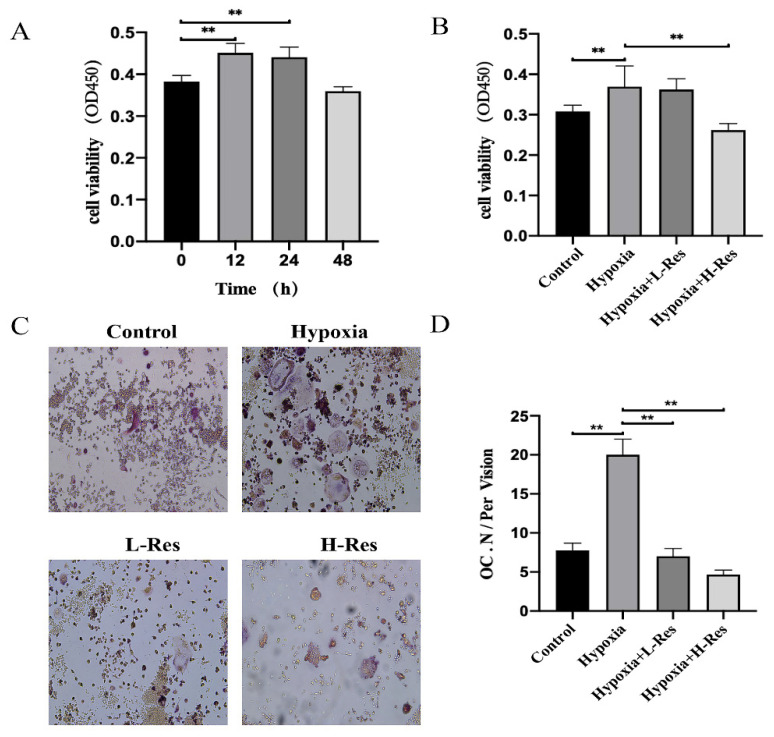
RES inhibited the cell viability and osteoclastogenesis in RAW264.7 under hypoxia. (**A**) The viability of RAW264.7 under hypoxia was measured. (**B**) The viability of RAW264.7 from control group, hypoxia group, L-RES group and H-RES group was detected. (**C**) TRAP staining results of RAW264.7 from control group, hypoxia group, L-RES group and H-RES group and (**D**) quantification. Values were expressed as mean ± SD. ** *p* < 0.01; all the assays were repeated more than three times.

Like osteoblastogenesis, osteoclastogenesis is also an important part of bone metabolism. Thus, the effect of RES on RAW264.7 was measured to evaluate its regulation of osteoclastogenesis under hypoxia. The CCK8 assay suggested that hypoxia could promote the proliferation of RAW264.7 at 12 h or 24 h, but started to inhibit such proliferation at 48 h (Figure 2A; *p* < 0.01). RES could inhibit this proliferation under hypoxia at 24 h at a high dose (Figure 2B; *p* < 0.01). To observe RES’ effect on the osteoclastogenesis under hypoxia, TRAP staining was used. Osteoclasts inducted from RAW264.7 were quantified. Hypoxia significantly increased the number of osteoclasts, but such an effect decreased after the treatment with RES in a dose-dependent manner (Figure 2C,D; *p* < 0.05).

### 3.3. RES Decreased Accumulation of ROS and HIF-1α in BMSCs under Hypoxia

**Figure 3:**
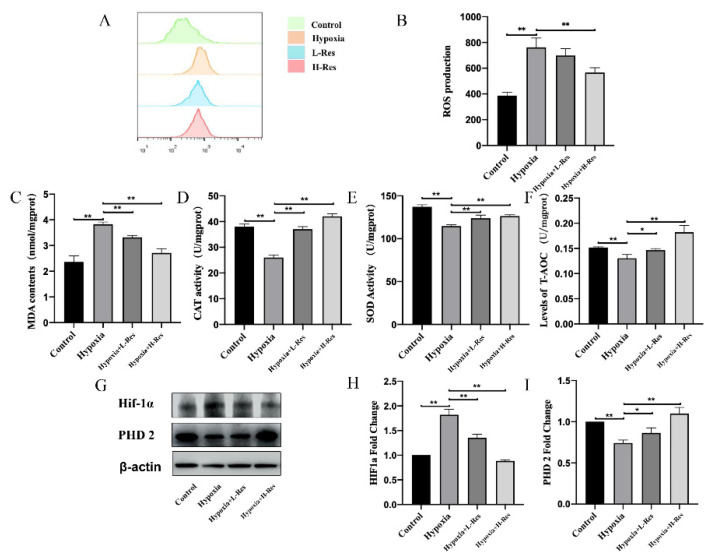
RES decreased accumulation of ROS and HIF-1α in BMSCs under hypoxia. (**A**,**B**) The level of ROS in BMSCs from control group, hypoxia group, L-RES group and H-RES group was detected and quantified. The content of (**C**) MDA and the activity of (**D**) CAT, (**E**) SOD, (**F**) T-AOC in BMSCs from control group, hypoxia group, L-RES group and H-RES group were determined. (**G**) The protein expression level of HIF-1α and PHD2 in BMSCs from control group, hypoxia group, L-RES group and H-RES group was determined and (**H**,**I**) quantified. Values were expressed as mean ± SD. * *p* < 0.05, ** *p* < 0.01; all the assays were repeated more than three times.

To explore the role of HIF pathways during the RES-mediated promotion of osteoblastogenesis in BMSCs, the ROS level, oxidative stress, and the expression of HIF-1α and PHD2 were detected. The results showed that hypoxia led to ROS accumulation, which could be attenuated by RES in a dose-dependent manner (Figure 3A,B; *p* < 0.05). Consistently, the content of MDA in BMSCs was enhanced by hypoxia, which could be attenuated by RES in a dose-dependent manner (Figure 3C; *p* < 0.05). On the other hand, the antioxidant such as CAT, SOD and T-AOC were inhibited under hypoxia, and the supplement of RES could reverse that inhibitory effect in a dose-dependent manner (Figure 3D–F; *p* < 0.05). At the same time, according to the results of western blotting, hypoxia promoted the accumulation of HIF1-α, whose amount got less after the supplement of RES (Figure 3G,H; *p* < 0.05). While the level of PHD2 was decreased under hypoxia, which was enhanced by RES in a dose-dependent manner (Figure 3G,I; *p* < 0.05).

### 3.4. RES Attenuated Bone Loss In Vivo under High Altitude Hypoxia

**Figure 4:**
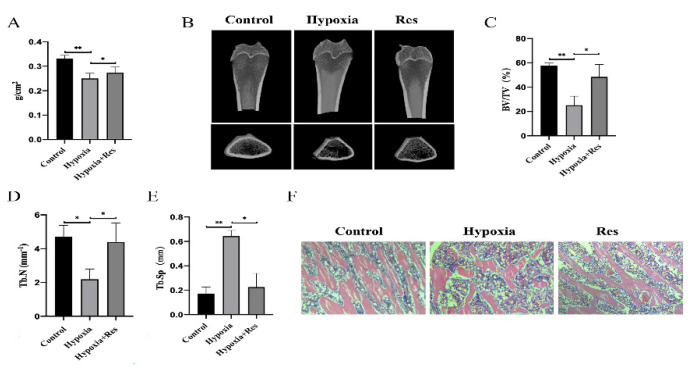
RES attenuated high-altitude hypoxia-induced osteoporosis in rats. (**A**) The BMD of rats from control group, hypoxia group and RES group. (**B**) Representative photograph of micro-CT reconstruction. (**C**) Bone volume per total volume (BV/TV). (**D**) Mean trabecular number (Tb.N). (**E**) Mean trabecular separation (Tb.Sp). (**F**) The results of HE staining of rats’ femurs from control group, hypoxia group and RES group. Values were expressed as mean ± SD. * *p* < 0.05, ** *p* < 0.01; all the assays were repeated more than three times.

Dual-energy X-ray absorptiometry is a commonly used method for measuring BMD. In our study, it was found that high-altitude hypoxia-treated rats had lower values of BMD than the control group, while the RES-treated rats had higher values of BMD than the hypoxia group (Figure 4A; *p* < 0.01). The microarchitecture of femurs was investigated via micro-CT examination before structural parameters of trabecular bone were quantified. Wistar rats showed a decrease in BV/TV and Tb.N and an increase in Tb.Sp compared to the control group. RES ameliorated bone loss, which is significant in the hypoxia group, and increased the values of BV/TV and Tb.Th and decreased the values of Tb.Sp (Figure 4B–E; *p* < 0.05). Additionally, HE staining was carried out to observe the morphology of bone tissue (Figure 4F). The trabecular bone arrangement of the rats exposed to hypoxia was disordered and sparse compared to the control group. The arrangement and distribution of trabecular bone in the RES group tended to be normal compared to the hypoxia group.

Notably, high-altitude and hypobaric hypoxia had an adverse effect on the growth and development of Wistar rats, and significantly reduced their body mass (*p* < 0.05). Rats in the RES group grew much better than in the hypoxia group, although there was no significant difference in body weight in the RES group (Table 1).

### 3.5. RES Attenuated Bone Remodeling Dysfunction In Vivo under High Altitude Hypoxia

**Figure 5:**
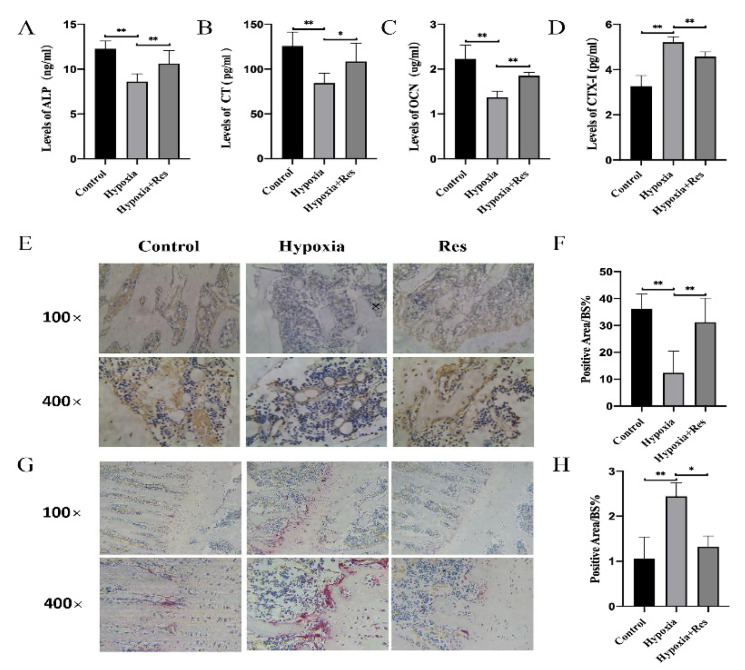
RES attenuated high-altitude hypoxia-induced bone remodeling dysfunction in rats. The serum level of (**A**) ALP, (**B**) CT, (**C**) OCN and (**D**) CTX-I in rats from control group, hypoxia group and RES group. (**E**) The immunochemistry results of RUNX2 staining of rats from control group, hypoxia group and RES group and (**F**) quantification. (**G**) The TRAP staining results of rats from control group, hypoxia group and RES group and (**H**) quantification. Values were expressed as mean ± SD. * *p* < 0.05, ** *p* < 0.01; all the assays were repeated more than three times.

To find out how RES elevated bone mass by osteoblastogenesis, the level of three bone formation markers, ALP, CT and OCN, were measured by ELISA. According to the results, the three markers decreased under hypoxia but RES treatment reversed that inhibitory effect (Figure 5A–C; *p* < 0.05). Immunohistochemical staining showed that high-altitude hypoxia-treated rats had a lower ratio of RUNX2-positive area than in the control group, but that ratio was increased by RES treatment (Figure 5E,F; *p* < 0.01). To investigate how RES elevated bone mass by osteoclastogenesis, the level of CTX-I was detected, and the number of osteoclasts was calculated by TRAP staining. The level of CTX-I also demonstrated the promotion of osteoclastogenesis under high-altitude hypoxia, which was reversed by RES (Figure 5D; *p* < 0.01). TRAP staining results demonstrated that the femurs from high-altitude hypoxia-treated rats had a higher ratio of TRAP-positive area than in the control group, and that RES lowered that ratio in the RES group (Figure 5G,H; *p* < 0.05).

### 3.6. RES Decreased Oxidative Stress and Accumulation of HIF-1α In Vivo under Hypoxia

**Figure 6:**
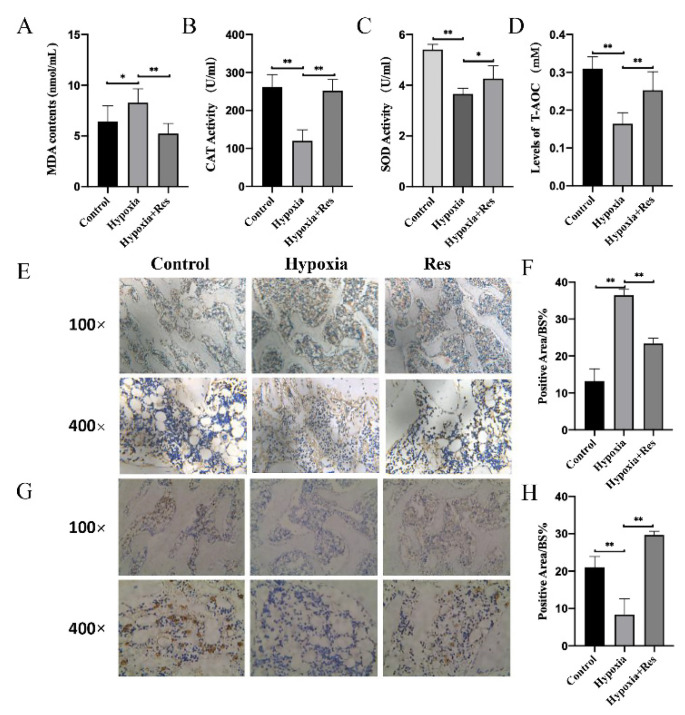
RES suppressed hypoxia-induced oxidative stress and the accumulation of HIF-1α in rats. The serum content of (**A**) MDA and the activity of (**B**) CAT, (**C**) SOD, (**D**) T-AOC in rats from control group, hypoxia group, RES group were determined. (**E**) Immunohistochemistry staining results of HIF-1α of rats from control group, hypoxia group, RES group and (**F**) quantification. (**G**) Immunohistochemistry staining results of PHD2 of rats from control group, hypoxia group, RES group and (**H**) quantification. Values were expressed as mean ± SD. * *p* < 0.05, ** *p* < 0.01; all the assays were repeated more than three times.

To investigate whether RES attenuated high-altitude hypoxia-induced osteoporosis in rats through the HIF-1α pathway in vivo, the oxidative stress and the expression of HIF-1α and PHD2 were measured. Consistent with the results in cells, the content of MDA in rats’ serum was enhanced by hypoxia, which could be attenuated by RES (Figure 6A; *p* < 0.05). On the other hand, the antioxidants, such as CAT, SOD and T-AOC, were inhibited under hypoxia, and the supplement of RES could reverse that inhibitory effect (Figure 5B–D; *p* < 0.05). Immunohistochemical staining was used to detect the in vivo level of HIF-1α and PHD2 in rats. Results showed that HIF-1α was accumulated in the rats under hypoxia, which was reversed significantly by RES (Figure 6E,F; *p* < 0.05). In contrast, PHD2 decreased in the rats under hypoxia, which was also reversed to a great extent by RES (Figure 6G,H; *p* < 0.01).

## 4. Discussion

Osteoporosis, as a systemic bone disease, greatly increases the risk of fracture. Nowadays, the research focus is on postmenopausal osteoporosis and senile osteoporosis. Some scholars have found that the etiology of postmenopausal osteoporosis is inseparable from the lack of estrogen [30]. Estrogen deficiency leads to osteoclast activation, resulting in the greater bone catabolism compared to the bone anabolism, which in turn leads to a decrease in bone density, finally [31]. Studies have shown that senile osteoporosis is caused by the aging of the body and organs, resulting in abnormal bone metabolism [32]. High-altitude people such as high-altitude workers rush into the high-altitude and are exposed to hypoxia for a long time, which seriously damages the bone health and leads to the occurrence of high-altitude osteoporosis in addition to the two well-known types of osteoporosis. The causes of osteoporosis in high-altitude areas are more complex than those of other types of osteoporosis, which may be related to such factors as low oxygen, high cold and ultraviolet radiation, acting on various systems of the body and causing lesions in human bones, digestion, circulation, cognition and memory [33,34]. Oxygen concentration is a crucial factor affecting bone metabolism, and prolonged hypoxia’s negative effect on bone metabolism has been confirmed [35]. High altitude hypoxia leads to heaps of free radicals and oxidative stress damage, which accounts for the disorder of bone metabolism in the body [36].

There are various treatments for osteoporosis. In addition to adjusting lifestyle and supplementing calcium, drug treatment is also essential. Anti-osteoporosis drugs include drugs that promote anabolism, inhibit decomposition, and some new drugs which are still under development [37]. Postmenopausal osteoporosis can be effectively treated with estrogen and estrogen-like drugs, but there is no clear specific drug for high-altitude osteoporosis. Antioxidative stress drugs may become one of the treatment strategies for high-altitude osteoporosis in response to the decrease of bone mineral density caused by high-altitude hypobaric hypoxia. RES, as a polyphenolic compound, is capable of directly scavenging a variety of oxidants, including hydroxyl radical (^●^OH), O_2_^●^¯, H_2_O_2_ and peroxynitrite. RES has the ability to affect the gene regulation of redox systems. [38]. Previous research by our group showed that RES could effectively relieve altitude-related diseases such as altitude polycythemia [29]. In this study, we evaluated the effect of RES as a safe medicine for long-term use in high-altitude osteoporosis using hypobaric hypoxia treated rats.

According to this study, the 1% hypoxic microenvironment inhibited the proliferation of BMSCs and significantly reduced calcium deposition and ALP activity after osteoinduction. At the same time, the expressions of some genes related to osteoblastogenesis trended down. RES treatment could effectively promote the proliferation and osteoblastogenesis of BMSCs under hypoxia. Stimulation of the osteoblastogenesis of BMSCs is the main criterion for evaluating the efficacy of anti-osteoporosis drugs. Animal experiments showed that, 9 weeks’ high-altitude hypobaric hypoxia significantly reduced femoral bone mineral density in rats, which is consistent with reports in the literature. After RES administration, the BMD and related bone formation markers’ level were significantly improved in vivo compared with the low-pressure and hypoxia treatment group, suggesting the beneficial effect of RES on high-altitude osteoporosis.

Tests on the oxidative stress state, quantification results of HIF-1α and PHD2 showed that RES treatment could reverse the oxidative stress, reduce the level of ROS and HIF-1α and enhanced PHD2 levels under hypoxia. HIF-1α is a transcription factor sensitive to oxygen concentration. Under hypoxia, the accumulation of HIF-1α leads to the expression of a series of genes [39,40]. Several studies have shown that bone metabolism is closely related to HIF-1α. It has been found that HIF-1α is able to promote bone formation, mainly due to the coupling of HIF-1α and VEGF, which can promote angiogenesis and cell glycolysis [41,42,43,44]. However, it is also argued that HIF-1α inhibits the bone formation-related transcription factor RUNX2 through the HIF-TWIST pathway, which is unbeneficial for bone formation [45,46,47]. In our experiments, HIF-1α increased under hypoxia, at the same time, the expression of RUNX2 and osteogenesis decreased, which is consistent with what was reported. PHD2 is a hydroxylase, and the hydrolysis of HIF-1α depends on its hydroxylation by PHD2. It is also reported that, under hypoxia, ROS affects the activity of PHD2, thereby affecting the accumulation of HIF-1α [48,49]. In addition, the ROS produced during iron overload can reduce the level of PHD2 and increase the accumulation of HIF-1α [50]. Therefore, according to our results, RES is helpful for the activity of PHD2 under hypoxia by scavenging ROS, thereby reducing the accumulation of HIF-1α, elevating RUNX2′s expression and promoting osteogenesis. However, there are still limitations to experiments. Although both cell and animal experiments showed that hypoxia promoted osteoclast differentiation and osteoclast activity, the mechanism related to osteoclasts in this experiment needs to be further explored.

**Figure 7:**
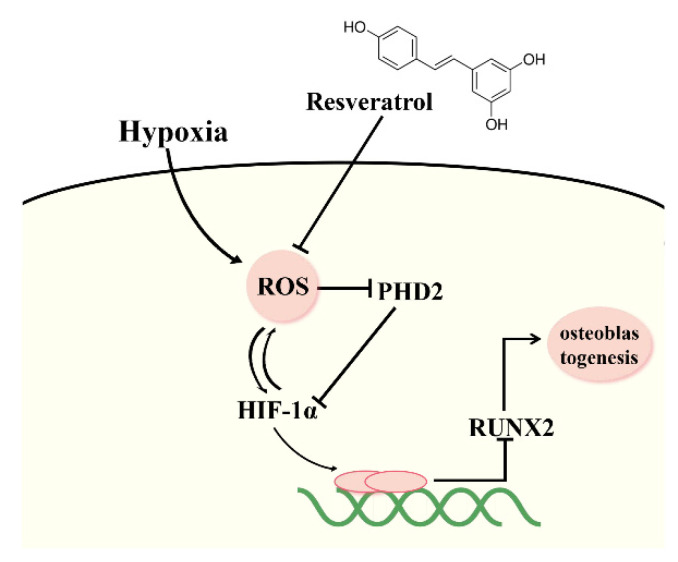
Mechanism of RES promoting osteogenic differentiation through ROS/HIF pathway. Schematic presentation of the role of RES on high-altitude hypoxia-induced osteoporosis via suppressing ROS/HIF-1α pathway. Under the condition of hypoxia, the release of ROS was enhanced, leading to reduced level of PHD2 and increased level of HIF-1α, which contributes to low expression of RUNX2 and deteriorative osteoblastogenesis. RES could effectively suppress the accumulation of ROS, thus rescuing the activity of PHD2, which leads to the downward level of HIF-1α and upward expression of RUNX2 and osteoblastogenesis.

In conclusion, as depicted in Figure 7, this study confirmed that RES could significantly alleviate high-altitude hypoxia-induced osteoporosis in rats by promoting bone anabolism and inhibiting bone catabolism, and this beneficial effect is related with the regulation of HIF-1. It is hoped that future research can shed more lights on the molecular mechanisms behind the regulation of RES on high-altitude hypoxia-induced osteoporosis.

## Figures and Tables

**Table 1:** Effect of oral administration of RES on body weight (g, $\bar{X}$ ± S).

Group	N	Initial	Metaphase	Final
Control	10	322.35 ± 13.88	393.01 ± 27.69	448.83 ± 32.35
Hypoxia	10	321.73 ± 12.95	293.17 ± 15.33 *	311.90 ± 22.29 *
RES	10	322.84 ± 12.29 ^NS^	287.98 ± 18.74 ^NS^	303.30 ± 23.47 ^NS^

* *p* < 0.05 was considered statistically different from control group; ^NS^ *p* > 0.05 not statistically different from hypoxia group.

## Data Availability

The datasets generated for this study are available on request to the corresponding author.
